# Population variability across geographical ranges: perspectives and challenges

**DOI:** 10.1098/rspb.2024.1644

**Published:** 2025-01-29

**Authors:** Cleber Ten Caten, Tad Dallas

**Affiliations:** ^1^ Department of Biological Sciences, University of South Carolina, Columbia, SC 29208, USA

**Keywords:** geographical range, population variability, performance curve, range position, geographical patterns

## Abstract

Populations fluctuate over time and across geographical space, and understanding how different factors contribute to population variability is a central goal in population ecology. There is a particular interest in identifying trends of population variability within geographical ranges as population densities of species can fluctuate substantially across geographical space. A common assumption is that populations vary more near species geographical range edges because of unsuitable environments and higher vulnerability to environmental variability in these areas. However, empirical data rarely support this expectation, suggesting that population variability is not related to its position within species geographical ranges. We propose that performance curves, which describe the relationship between population growth rates and environmental conditions, can be used to disentangle geographical patterns of population variability. Performance curves are important for understanding population variability because populations fluctuate more in locations where they have lower growth rates owing to unsuitable environmental conditions. This is important for the assessment of these geographical patterns in population variability because geographical edges often do not reflect environmental edges. Considering species performance curves when evaluating geographical patterns of population variability would also allow researchers to detect populations that are more susceptible to future changes in environmental conditions.

## 1. Introduction

A major goal in population ecology is to understand spatial and temporal patterns of population variability [1–3]. Understanding population variability is important because more variable populations are at greater risk of going extinct because of the dramatic changes in peaks and crashes that such populations exhibit [4,5]. Considering population variability patterns has become an important component to aid conservation efforts [5,6], and there is a great interest in identifying the main drivers of population variability across different species. Population variability is affected by a myriad of intrinsic (e.g. demographic stochasticity and life-history traits) and extrinsic (e.g. fluctuations in environmental conditions and patch quality) factors [1,7,8], where the importance of such factors in driving population variability likely varies across different populations.

**Figure 1.**
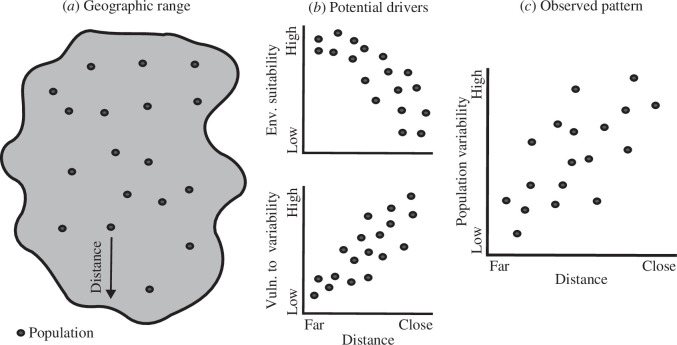
Populations occurring closer to the edge of a species geographical range (*a*) are assumed to be exposed to less suitable and to be more vulnerable (Vuln.) to variable environmental (Env.) conditions (*b*), which would decrease growth rate and lead to more variable populations in those areas (*c*) owing to smaller population densities and demographic stochasticity. These are some of the mechanisms that could explain the putative relationship between population position within the species geographical range and population variability.

A long-held hypothesis is that populations are more variable near the edges than near the centre of species geographical ranges [9,10]. There is an expectation that sites occurring near range edges have poorer environmental conditions than sites occurring near the range centre, making populations found near geographical range edges more vulnerable to environmental variability [11,12]. This would decrease population growth rates, leading to smaller population sizes that are more variable owing to demographic stochasticity being more prevalent in these populations (figure 1). A reduced genetic diversity near geographical range edges could further contribute to an increase in population variability owing to the lower adaptive potential of these populations [11,13]. Moreover, edge populations are often assumed to be more isolated, and less likely to receive immigrants through dispersal events, which could also affect the variability of these populations [6]. This could occur through meta-population dynamics, where edge populations are sometimes assumed to be sink populations that cannot exist without continued immigration from source populations [14,15]. Despite these clear predictions that edge populations should be more variable than central populations, few studies have assessed these trends [12,16]. This likely stems from the challenging need of continuously sampling several populations across broad spatial extents to evaluate patterns of population variability within species geographical ranges, representing an important gap in our understanding of geographical patterns of population dynamics [12,17].

Environmental conditions influence population dynamics such that vital rates and resulting population densities fluctuate more in variable than in constant environments [2,18]. The effects of environmental conditions on population dynamics can be evaluated with performance curves, which describe the relationship between population growth rates and environmental conditions. Performance curves have been used to predict extinction risk, geographical range dynamics and species responses to climate change [19–22], while their use to examine differences in local population dynamics across geographical ranges remains unexplored. We propose that performance curves provide a valuable framework for understanding population variability across geographical ranges because populations fluctuate more in locations where they have lower growth rates as a result of unsuitable environments. Performance curves provide a direct link to how changing environmental conditions influence population growth rates and the population dynamics of a given species. This allows the simultaneous evaluation of how species-specific environmental responses and changing environmental conditions interact to shape geographical patterns of local population dynamics. This framework addresses a key assumption, that environmental conditions are poorer near range edges, that is often made when evaluating geographical patterns of population dynamics [12,23]. Moreover, performance curves allow the identification of populations that are more susceptible to future climate change as environmental conditions will become unsuitable in parts of species geographical ranges [22,24].

We first provide a brief overview of some of the intrinsic and extrinsic factors that affect population variability and we synthesize the current evidence on population variability patterns across species geographical ranges. Intrinsic and extrinsic factors are defined as biological and non-biological components, respectively, that affect population growth rates and population dynamics. We use simulations to show that performance curves are useful to understand population variability and that having performance curves with different properties leads to contrasting geographical patterns of population variability across species. Lastly, important next steps in the study of spatial patterns in population variability are outlined.

## 2. Intrinsic drivers of population variability

### (a) Species traits

Species traits are important factors that affect population dynamics. Body size is a key trait that influences population dynamics, such that species that have small body sizes tend to have more variable populations than species with larger body sizes [25,26]. This occurs because species with small body sizes often have high population growth rates that can cause an increase in population variability through overcompensating density dependence when these species have non-overlapping generations [26,27]. The dispersal ability of species could also impact population variability given that the dispersal of individuals between populations affects population variability [28]. Theoretical models predict that lower dispersal increases population variability, but experimental evidence supporting this expectation is limited [28,29]. This indicates that different traits can potentially influence the patterns of population variability that species have.

### (b) Species interactions

Species interactions can affect population dynamics because sites have limited resources that can be used by individuals of different species [30,31]. The type of interaction between two species can determine its effects on growth rates, such that mutualistic interactions have positive effects on population growth rates while antagonistic interactions negatively affect population growth rates [32,33]. The strength of interactions, alongside the type of interaction, affects population dynamics such that strong interactions can lead to more or less variable populations, depending on whether species are exhibiting mutualistic or antagonistic interactions [34,35]. However, changes in abiotic and biotic conditions across geographical space or over time can modify the effects of species interactions on population dynamics [36,37], suggesting that the level of interaction specialization [1] and local environmental conditions can be important to determine population variability.

### (c) Demographic stochasticity

Demographic stochasticity describes fluctuations in populations in a constant environment because of random events related to birth and death of individuals in a population [8,38]. Two of the drivers of demographic stochasticity are stochastic sex determination and demographic heterogeneity. Stochastic sex determination leads to imbalances in the sex ratio of a population, which often increases the variance of population growth rates, whereas demographic heterogeneity relates to differences in birth and death probabilities of individuals of different sizes [8,39]. The effects of demographic stochasticity are more prominent in small populations, where it can often be the main driver of temporal variability in population densities [7,8]. Small populations usually have more variable growth rates [40], and species with small population densities often have more variable populations than species with larger population densities [9,41], likely because of demographic stochasticity.

## 3. Extrinsic drivers of population variability

### (a) Environmental variability

Fluctuations in environmental conditions are important extrinsic factors affecting population variability [8,42]. Sources of environmental variability can be periodic, stochastic or catastrophic. Periodic environmental variability refers to predictable changes in environmental conditions, such as seasonal differences in temperature [43]. Stochastic environmental variation occurs through unpredictable sources of variability in the environment, and catastrophic sources of variability are rare extreme events, such as hurricanes, that significantly affect populations [43]. Here we use environmental variability to refer to stochastic environmental variations. More variable environments can increase population variability because of demographic constraints caused by extreme environmental conditions [42,44]. Variation in environmental conditions similarly affects all individuals in a population, leading to decreases in long-term growth rates and more variable population dynamics [8,45,46]. Environmental variability can have a stronger impact on population dynamics than changes in average environmental conditions [47,48], but environmental variability can also increase long-term growth rate, depending on the shape of the species performance curve [49]. This suggests that knowledge about species performance curves is essential to understand how environmental variability affects population dynamics.

### (b) The geography of occupied patches

The spatial structure of patches where populations occur affects population density [50]. Small and isolated patches often have smaller and more variable populations than larger and well connected patches [51]. Small patches have smaller populations because of the limited space and resources available for species in these locations, which reduce population growth rates [52–54]. This can amplify the effects of demographic stochasticity in populations found in these areas. Individuals are less likely to migrate to highly isolated patches, such that this decrease in dispersal is often linked to more variable population dynamics [28,55]. Thus, an interplay between patch area and isolation can lead to differences in population dynamics across geographical ranges.

### (c) The possible interaction between different drivers of population variability

Population variability might be driven through an interplay between intrinsic and extrinsic factors. For example, both demographic stochasticity and fluctuating environmental conditions can similarly drive population dynamics, depending on the size of the populations [8]. Increasing levels of environmental variability lead to contrasting population dynamics between interacting species when they respond differently to environmental conditions [46], further exemplifying the interaction between different mechanisms driving population variability. This highlights that the assessment of population variability patterns across geographical space can be a complex task as multiple mechanisms might interact to drive such dynamics.

## 4. Gauging support for geographical patterns of population variability

We conducted literature searches on Web of Science (WoS) to obtain articles that evaluated population variability patterns across species geographical ranges. We performed our search using individual keywords combined with Boolean operators (restricting or expanding the search using ‘OR’ and ‘AND’), and asterisks to consider multiple words that have different endings. Our search was performed on 6 February 2024, using the following search terms: ‘population varia*’ OR ‘population stability’ AND ‘geographical range’ OR ‘range position’. We restricted our search to the field Ecology, resulting in 1302 articles. We filtered this initial list to keep articles that compared temporal trends of population variability across different parts of species geographical ranges by screening article titles and abstracts. Studies that compared trends of population variability across species or at the community level were not considered. This resulted in a final pool of five studies from our WoS search. We checked the references and articles that cited these five studies to include studies that were not returned in our search, resulting in four additional studies being added to our list (table 1).

**Table 1.** List of articles that were found in our Web of Science search. Variability estimates observed were the coefficient of variation (CV), standard deviation (SD), variance (var), index of density variation (*s*), amplitude, and the standard deviation of residuals. Predictors (Predictor) representing the geographical range position of populations were often continuous (cont.), but there was a case of a discrete (disc.) predictor being considered. The number of species (*n*) considered in each study ranged from 1 to 24, with support for these relationships being variable. sig. *n* and dir. represent the number of species where population variability was significantly predicted by geographical range position and whether populations are more (+) or less (−) variable near geographical range edges.

variability estimate	predictor	no. species, *n*	sig. *n* and dir.	region	reference
CV	latitude (cont.)	24	6(+)	Great Britain	[56]
s.d.	—	—	7(+)	—	—
log(CV)	distance to edge (cont.)	6	2(+)	North America	[17]
s.d.(log_10_*n*)	geographical score (cont.)	1	1(+)	Japan	[57]
amplitude	—	—	—	—	—
CV	density (cont.)	3	3(+)	North America	[3]
CV	site northing (cont.)	19	4(+)	Great Britain	[58]
s.d.(log*n*)	—	—	2(+)	—	—
s.d.(log*n*)	elevation (disc.)	1	1(+)	Japan	[59]
var(log*n*)	latitude (cont.)	8	5(−)	North America	[60]
CV	latitude (cont.)	2	2(+)	Fennoscandia	[61]
*s*	—	—	—	—	—
s.d.(residuals)	latitude (cont.)	1	0	North America	[62]

Despite being considered ‘conventional wisdom’ [63], we found that the relationship between population variability and geographical range position has been severely under-assessed compared with geographical trends in population density or genetic variability [12,16]. We observed taxonomic biases in the studies we found, where population variability was often evaluated for animal species, and we did not find studies considering plant species. This lack of studies using plant species likely stems from the fact that most assessments of variability in plant population dynamics consider the variability of demographic parameters (e.g. recruitment and survival) across species geographical ranges [64,65] rather than variability in population densities. As demographic parameters might vary in different directions across plant species geographical ranges (i.e. demographic compensation [65–67]), and it is unclear how the contrasting geographical trends of variability in these demographic parameters would affect spatial patterns of population variability, we did not consider these studies. We acknowledge that some studies have evaluated the variation of population growth rates across geographical ranges of plant species [68–70], which could be used as a proxy for population variability. We did not consider these studies in our analyses because these population growth rates were often estimated through matrix models that make assumptions, such as equal survival and fecundity of adults derived from seeds of different ages, that are challenging to consider in our assessment.

The majority of the studies evaluating patterns of population variability considered terrestrial species and occurred in the Northern Hemisphere (table 1). The most often used estimates of population variability were the coefficient of variation (CV) and the standard deviation (s.d.) of population densities. Alternatively, there were different estimates that were used to represent the position of populations within species geographical ranges. Latitude was the most commonly used estimate of geographical range position [56,60–62]. Using latitude as a proxy for position within the species geographical range might be inadequate because populations should be more variable near geographical range edges regardless of latitude. Two populations that are found at the same latitude within the species geographical range could have different geographical range position, because they are found at different longitudes, and would supposedly exhibit contrasting patterns of population variability. However, considering latitude as a proxy for geographical range position is understandable given that several environmental conditions that are thought to be major drivers of population variability might exhibit a latitudinal gradient [61]. Other estimates of range position included distance to geographical range edge [17], geographical scoring [57], site northing [58], elevation [59] and population density [3].

Overall, the four studies that found support for the occurrence of more variable populations near geographical range edges considered fewer than five species [3,57,59,61], although a lack of relationship between range position and population variability was also reported in a study that considered one species [62]. Conversely, three studies that considered over five species often found support for the occurrence of more variable populations near geographical range edges for only approximately 30% of the species considered [17,56,58], and in one study opposite patterns (i.e. less variable populations near range edges) were observed [60]. This prevalence of 30% of the species exhibiting geographical patterns of population variability is similar to the observed prevalence of geographical patterns of population density [12,23], suggesting that trends of population density and population variability within geographical ranges are not common. Nonetheless, it still remains an open question whether the same species show similar patterns of population density and population variability across geographical ranges given that these trends are often evaluated separately from each other, and there is an expectation that similar mechanisms drive population variability and density across geographical ranges [23].

Our search shows that most of the support for the expectation that species have more variable populations near their geographical range edges comes from studies that evaluated these patterns for small sets of species, while there is a lack of studies evaluating these relationships for larger sets of species, representing an important knowledge gap in our understanding of population dynamics across species geographical ranges. The data needed to evaluate these trends of population variability across geographical ranges need to cover a broad spatial and temporal scale, being a clear limiting factor when addressing these questions. These challenges can be overcome through the use of large-scale monitoring programmes that continuously record species occurrence and abundance at different locations (e.g. Breeding Bird Survey and the United Kingdom Butterfly Monitoring Scheme), or community-maintained databases that summarize collection of assemblage data worldwide (e.g. BioTIME and sPlotOpen), or through the use of citizen-science databases (e.g. eBird and iNaturalist). However, spatial biases in sampling and in over-representation of species with specific traits may need to be addressed before using citizen-science databases to answer questions on population dynamics across species geographical ranges [71,72].

## 5. Why are patterns of population variability across geographical ranges rarely observed?

Patterns in population variability across species geographical ranges have not been widely evaluated, and we found limited evidence supporting the occurrence of such trends. This lack of support for this expectation could be explained by different factors. For example, it is assumed that species are in equilibrium and that environmental conditions gradually deteriorate towards the edges of species geographical ranges, ultimately leading to more variable population dynamics in these regions. While several environmental conditions exhibit latitudinal gradients [73], it is unclear why environmental conditions would gradually become unsuitable towards all edges of species geographical ranges. Variables affecting population dynamics could operate at a finer spatial scale than studies often consider and have low spatial structure [10,12]. This can cause heterogeneity in landscapes and influence the spatial signal in how population variability is structured across geographical space. For example, landscape heterogeneity caused by topographic variability can provide suitable microclimatic conditions near edges of geographical ranges that can reduce population variability in these locations [74,75].

Populations occurring in different parts of species geographical ranges can have different types of adaptations to local environmental conditions that stabilize population dynamics across their geographical ranges [76]. For example, populations of plant species occurring across gradients of aridity might show specific local adaptations in morphological leaf traits that improve their performance when occurring in unfavourable conditions [77,78]. These adaptations to different environmental conditions across geographical ranges can involve several traits that are associated with different aspects of population performance [79,80]. This can lead to the occurrence of demographic compensation across geographical ranges (e.g. a higher death rate in a given location can be offset by increased fecundity of that population) and lead to similar patterns of population variability across geographical ranges [66,67]. Although not all species show demographic compensation [65,81,82], demographic compensation could explain the lack of population variability patterns across geographical ranges of most species.

Populations can be more or less variable depending on their position within the species thermal performance curve and the amount of environmental variability that they experience [49,83]. For example, populations found in warmer locations are more susceptible to temperature fluctuations independent of their position within the species geographical range [84,85]. This indicates that position of a population within the species thermal performance curve might be a more important predictor of population variability than geographical range position. Populations can also exhibit local adaptations in their thermal performances such that populations found in warmer environments can be more tolerant to warming conditions [86] and populations found in the colder environments could be more tolerant to lower temperatures [87]. Although species performance curves are often described in terms of thermal performance along temperature gradients, other environmental conditions might be more important to determine population dynamics of a given species [88]. This suggests that environmental conditions and the position of a population within the species performance curve, and not geographical range position, are key factors driving population variability across geographical ranges.

## 6. The importance of the shape of species performance curves

Population dynamics could be better understood based on the position of populations within species niches rather than based on their position within geographical ranges [89,90]. This links how species niche requirements affect their geographical distribution [91] and addresses some of the issues with using geographical range position as predictor of population dynamics. However, the niche describes the set of environmental conditions needed for a species to persist indefinitely in an area [92], suggesting that using the niche to evaluate population dynamics might be challenging. For example, a species may have a niche that allows it to persist in locations that have temperatures between 8 and 30°C. Determining how this species population dynamic changes along this temperature gradient based on the niche is difficult as the relationship between population growth rate and temperature can have different shapes that would lead to contrasting patterns of population dynamics along this temperature gradient.

Performance curves have been used to understand the effects of environmental conditions on population growth rates and how they affect extinction risks and limit species geographical distributions [21,22]. Spatial differences in environmental conditions affect population dynamics such that performance curves can also be used to elucidate patterns of population variability that are observed within the geographical ranges of different species. We show that differences in the shape (i.e. the level of symmetry) that species exhibit in their performance curves [93] are sufficient to lead to contrasting patterns of population variability across their geographical ranges even when their performance curves have the same optimum (see box 1). This occurs because some environmental conditions have different effects on the population dynamics of species with performance curves of different shapes. These results show that performance curves are important determinants of species differences in patterns of population variability across geographical ranges in addition to limiting population growth rates and geographical distributions [21,22]. Considering species performance curves allows a disentanglement of geographical population variability trends according to the environmental conditions that populations are being exposed to in a given area. This is an important step towards understanding spatial patterns of population variability as geographical and climatic edges are not concordant for most species [94,95].

Box 1. **The influence of species performance curves on geographical patterns of population variability.**

Species performance curves often describe the relationship between population growth rate and environmental conditions. Considering the shape of performance curves can be crucial to understanding patterns of population variability within species geographical ranges. Species performance curves can have different shapes (e.g. symmetric and asymmetric [93]), which can be enough to affect patterns of population variability within species geographical ranges, and might explain why species exhibit different geographical patterns of population variability.

**Figure 2.**
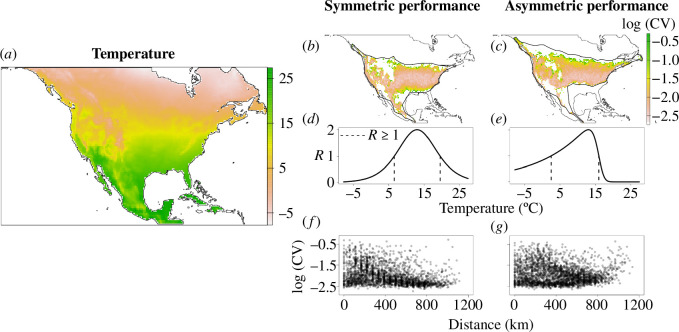
North American landscape (*a*) used to simulate population dynamics of the species with symmetric (*b,d,f*) and asymmetric (*c,e,g*) performance curves. In both curves, performance is maximized at a value of 13 and the dashed black lines represent the range of environmental conditions in which populations have growth rates ≥1 (*d,e*). The species with a symmetric curve (*b,f*) had more variable populations near geographical range edges (thicker black lines in panels (*b*,*c*)) while this relationship was much weaker for the species with an asymmetric performance curve (*c,g*).


To demonstrate the influence of the shape of the performance curve on geographical patterns of population variability, we simulated the population dynamics of two species that have performance curves with different shapes across the same landscape. We used North America as landscape (7403 cells), and we considered mean annual temperature, ranging from *ca* −8°C to *ca* 27°C , as the environmental condition that affected our virtual species (figure 2*a*). The population dynamics of both species were modelled following a discrete time Ricker model, where populations grow according to rate *R* and intraspecific competition (α = 0.005) limits population size *N*. The number of offspring obtained in each generation is estimated from a Poisson distribution with mean *R*, and survivorship at each time step is defined by a binomial random variable with probability ${\text{e}}^{-\alpha N_{t}}$. These two processes represent the main drivers of demographic stochasticity in this model. Note that in this model there is no dispersal occurring between the different populations.

(6.1) $$N_{t+1}=N_{t}\;R\;{\text{e}}^{-\alpha N_{t}}.$$

The *R* of a population in a given location was estimated based on the performance curve of each species. The main difference between the species performance curves is their shape, where one species has a symmetric and the other has an asymmetric performance (figure 2*d,e*). Both species have the same optimum environmental condition (13°C), and the area under the curve for which populations are able to persist indefinitely (i.e. *R* ≥ 1) is also the same for both species. This illustrates a case where species achieve their highest performance under similar environmental conditions, but that their performance deteriorates at a different rate away from this optimum.

We started the simulation with every cell in the landscape being occupied by 50 individuals, and we ran the models for 200 generations. We calculated the coefficient of variation of abundance to estimate population variability for all cells that did not go extinct between generations 100 and 200. We assessed the relationship between population variability and distance to the geographical range edge using Spearman’s correlation to account for potential nonlinearities in these relationships. The species with an asymmetric performance curve occupied *ca* 11% more cells than the species with a symmetric curve, and it also had, on average, slightly less variable populations across the landscape (figure 2*b*,*c*). The relationship between variability and distance to geographical range edge was influenced by the shape of the performance curves. The species with a symmetric performance curve tended to have more variable populations near range edges (ρ = −0.38, *p *< 0.01; figure 2*f*) while this relationship was much weaker for the species with an asymmetric performance curve (ρ = −0.07, *p *< 0.01; figure 2*g*). This highlights that differences in the shape of species performance curves affect geographical patterns of population variability even when species achieve their highest performance under similar environmental conditions.

The occurrence of dispersal events between populations can strongly influence observed geographical patterns of population variability. In our simulation, population variability was only estimated for sites where the species had *R *≥ 1. This was done to isolate the effects of the shape of species performance curve on population variability across geographical space, but it is a simplified representation of population dynamics. By allowing dispersal of individuals between sites, the occurrence of source–sink dynamics becomes possible, where populations that are sinks (i.e. they have *R* < 1) can be maintained through continuous dispersal from source populations. This continuous dispersal of individuals between source and sink sites could have direct impacts on trends of population variability as dispersal can increase or decrease population variability [28,29].

The level of spatial autocorrelation that environmental variables show can further influence geographical patterns in population variability. Spatial autocorrelation varies across different environmental conditions (temperature and precipitation) and regions (lowlands and mountainous regions), which can affect landscape heterogeneity and population dynamics. Highly spatially autocorrelated environmental conditions can lead to more pronounced geographical patterns of population variability compared with environmental conditions with low spatial autocorrelation. Thus, an interplay between the shape of species performance curves and the level of spatial autocorrelation of the environmental conditions that affect species performances is likely to drive geographical patterns of population variability.

## 7. Potential effects of changing environmental conditions on population variability

The characteristics of species thermal performance curves vary across geographical space. Species occurring in tropical regions often have narrower performance curves and occur in areas that have environmental conditions closer to their optimum [24]. This suggests that tropical species might be more susceptible to changes and fluctuations in environmental conditions as they are closer to their maximum critical temperature than are temperate species ([96–98], but see [48]). This has prompted an interest in using performance curves to predict how climate change and environmental variability will affect species [19,22,24,97,98]. Overall, environmental variability is predicted to decrease species performance [24,98], and species occurring in mid latitudes might be particularly susceptible to changes and variation in environmental conditions [93,97]. Even small changes in the mean and in the variability of environmental conditions can be detrimental for tropical species [24,98], while temperate species are exposed to higher environmental variability, which also negatively impacts their performance [20,97]. This shows that changing environmental conditions can negatively affect species performances in different ways depending where species occur.

Here, our goal is to demonstrate that species performance curves can also be used to understand patterns of population variability in constant and variable environments and how that can influence geographical patterns of population variability. We simulated population dynamics of two hypothetical species following the same framework described in box 1, but we considered two different scenarios. In one scenario, population dynamics were simulated in a constant environment where environmental conditions did not vary from generation to generation. In the other scenario, we simulated an instance where there is environmental variability occurring from generation to generation following a normal distribution N∼(0,3), and we assumed that populations would respond immediately to these fluctuations in environmental conditions. If environmental variability caused a population to go extinct at some point before the end of the simulation, we calculated population variability considering the individuals up until the extinction occurred.

**Figure 3.**
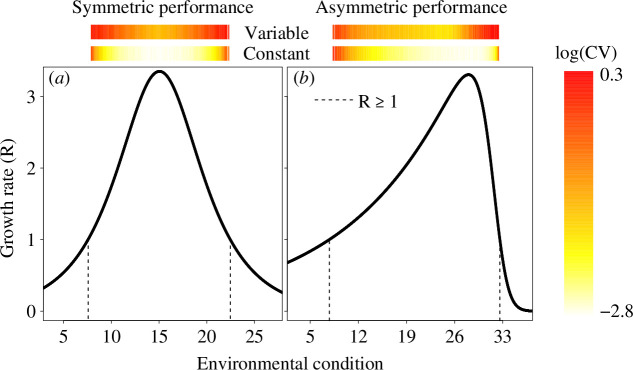
Population dynamics were simulated for 200 generations for species with symmetric (*a*) and asymmetric (*b*) performance curves for populations occurring along an environmental gradient. The coloured bars on top of the figures represent population variability estimated through the coefficient of variation (log(CV)) considering generations 100–200 for constant (Constant) and variable (Variable) environments. Populations occurring near the edges of the performance curves (areas near the dashed lines where population growth rate is *ca* 1) are more variable than populations found where they have high performance, especially when environments are constant. When environments vary over generations, even populations occurring in environments where they have on average high performance vary significantly more when compared with constant environments.

Some populations vary more than others because of their position within the species performance curves (figure 3). Populations found in less suitable environmental conditions (i.e. in environmental conditions where growth rate is *ca* 1) are naturally more variable regardless of the performance curve shape when environmental conditions are constant (figure 3). This occurs because these populations have lower growth rates, and demographic stochasticity becomes an important driver of population variability in these cases. This suggests that as mean climatic conditions change over time, patterns of population variability within geographical ranges will also change. Variability in environmental conditions is predicted to increase with climate change [24,48], and in these cases population variability will increase especially for populations found near optimum conditions (figure 3). These results show that decreases in performance caused by environmental variability [24,98] are reflected in the higher variability of population densities. This is an example of Jensen’s inequality, where, despite average environmental conditions being the same in the constant and variable scenarios, fluctuating environments lead to more variable populations because of the disproportionate effects that positive and negative values of environmental variation have on growth rates [47,48]. This suggests that environmental variability can obscure patterns of population variability across geographical ranges particularly for species that occur in areas with high environmental variability (e.g. temperate regions).

Although our simulations were not spatially explicit (i.e. there was no dispersal occurring between populations), our results indicate that coupling information on species performance curves and environmental variability can improve our understanding of geographical patterns of population variability. This framework addresses the problem that populations found at edges of geographical ranges can be occurring in optimum environmental conditions [81,94], and could explain the lack of consistent geographical patterns of population variability across different species. Future changes in mean environmental conditions, alongside increased environmental variability, will contribute to form complex geographical patterns of population variability as this will reshuffle the position of populations along species performance curves.

## 8. Moving forward on the evaluation of geographical patterns of population variability

Describing geographical patterns of population variability is challenging because of the multiple intrinsic and extrinsic factors that cause fluctuations in population densities. Predictions of population variability based on geographical range position are appealing for their simplicity, but they have several limitations because of their simplifying assumptions, such as that geographical ranges are static and that environmental conditions are constantly unsuitable in range edges. Shifts in species geographical distributions and population dynamics at different parts of their geographical ranges because of ongoing changes in environmental conditions challenge these assumptions [81,85,99]. Dispersal and colonization processes affect population dynamics and the ability of species to occupy suitable environments across geographical space. This occurs, for example, for species that are invading new areas [100], further highlighting the limitations of using geographical range position as a predictor for population variability. Performance curves overcome these problems and address assumptions that environmental conditions are spread in a specific way across geographical ranges and that ranges are static. Performance curves have been used to predict current and future geographical distribution of species [21,22], and we show that they are also useful to understand geographical patterns of population dynamics.

Using performance curves allows one to disentangle geographical patterns of population variability, but there are some potential drawbacks with the use of this approach. Information on performance curves is not readily available for several species, and most of the research on this topic has been focused on thermal performance curves of ectotherms [21,24,48,96,98] while knowledge on endotherms’ and plants’ performance curves is generally lacking. This could pose some constraints on the use of performance curves to understand geographical patterns of population variability, but this limitation could be eventually overcome as descriptions of endotherms’ and plants’ performance curves become more widely available. Recent modelling frameworks are able to predict species performance curves from response trait data rather than from population-level information [101]. Such frameworks can be used to address knowledge gaps for species where trait response data are more broadly available, but for which we have limited knowledge on their performance curves. Another potential limitation of our approach is that we define performance curves at the species level, which may not be able to capture local adaptations exhibited by different populations [86,102]. This problem can be overcome by sampling individuals from different populations, to account for potential local adaptations, when describing species performance curves. Below we outline some important next steps that could be considered in the study of geographical patterns of population variability:

1. How does dispersal affect geographical patterns of population variability? Theoretical models predict that dispersal should reduce population variability, but empirical evidence suggests that the effects of dispersal on population variability are more complex.
2. How do microclimatic conditions affect population variability trends across geographical ranges? Coarse environmental predictors are often used to assess the role of environmental conditions on population dynamics, but microclimatic conditions can provide suitable environments and buffer population variability in areas that are seemingly unsuitable for a species.
3. What is the role of biotic interactions in shaping geographical trends of population variability? Are the effects of antagonistic and mutualistic interactions on population variability similar in different parts of species geographical ranges?
4. What is the influence of temporal and spatial sampling scale on geographical patterns of population variability? Different patterns of population variability could be observed depending on how abundance data are aggregated in geographical space and how long populations have been sampled.
5. How does periodic environmental variability affect population variability across geographical ranges? Seasonal fluctuation in environmental conditions can lead to variation in populations because of seasonal demography that species can exhibit.
6. How do differences in life-history traits affect geographical patterns of population variability for different species? Is there a relationship between intraspecific variability of life-history traits and population variability across geographical ranges?
7. Is the geography of occupied sites important to determine geographical patterns of population variability? Population variability across geographical ranges could be explained by the size and the amount of resources of the sites where populations occur.
8. Are there taxonomic differences in how populations vary across geographical ranges? Some taxa can have more or less variable populations across geographical ranges because of their ability to track suitable environments across geographical space.
9. How is global change influencing population variability across geographical ranges? Species are shifting their geographical ranges because of changing environmental conditions, but it is unclear how population variability of these species is being affected by global change.
10. How do different factors interact to drive geographical patterns of population variability? For example, changes in environmental conditions can modify the effects of species interactions on population dynamics, highlighting the importance of considering how different drivers of population dynamics might interact across geographical space to regulate geographical patterns of population variability.

geographical patterns of population variability have often been evaluated using observational data. However, the use of theoretical models and controlled experimental approaches is needed to further our understanding of geographical patterns of population variability. For example, the role of species interactions or dispersal on spatial patterns of population variability can be challenging to assess with observational data, but these factors can be more easily considered in simulation studies and mesocosm experiments. A thorough understanding of geographical patterns of population variability will be obtained when theoretical models are coupled with experimental approaches and observational data to fully disentangle the role of different factors in driving geographical patterns of population variability.

## Data Availability

R code to reproduce the simulations are available on Figshare at [103].
